# Care of Babies

**Published:** 1869-10

**Authors:** 


### CARE OF BABIES.

Poor babies! How we pity them. Unable to indicate the locality or nature of their pains, or to defend themselves against the attacks of their tormentors, they can but cry and suffer, while the affectionate mother or impatient nurse tries to relieve its aches by means of the most approved baby remedies. "Mrs. Winslow's"—"Mrs. Wheeler's"—and "Mother Bailey's Soothing Syrups"—are fed the innocent little creatures by the quart. They are trotted, churned, and rocked—bandaged, smothered, and stuffed, until they fall to sleep from sheer exhaustion

They are subjected to all manner of torture, which the kindly disposed nurse administers by virtue of her fallacious ideas of kindness. One of the most nonsensical and injurious practices to which the baby is subjected, is that of bandaging. A piece of flannel, as broad as your two hands, is pinned about the abdomen of the babe, and drawn as tightly, usually, as the strength of the nurse will permit. This is done for the purpose, they will tell you, of preventing the child from "hurting itself" when crying. Wonderful invention! But, is it not strange that Nature did not provide all babies with little bellies sufficiently strong to endure any amount of crying? Surely, if she has presented us with such an imperfect specimen of her work, she should at least furnish us with the bandage! Therefore, why was not this necessary implement born with the baby? What a singular oversight, to be sure! But seriously, this habit of bandaging babies is one of the most frequent causes for their illness and crying. It compresses the lungs by throwing the bowels and stomach against the diaphragm, preventing its action, so that pain is actually caused in attempting to inflate the lungs. It causes constipation, indigestion and vomiting, from pressure upon the bowels and stomach—it prevents proper circulation in the bowels and through the skin. In short, it is unnatural, unnecessary and hurtful, therefore should be abandoned. Let the clothing be of fine flannel and fit baby loosely—let its abdomen and chest have free play, and no amount of crying can do its little belly harm.

Many blunders are made in the means of nourishing a baby. If a baby is properly fed, and its food agrees with it, it will gain in weight every week, and become fat and plump. But should it lose in flesh, become spare and thin, and its skin assume a bluish tint—should it suck greedily and remain unsatisfied, be sure the food is not suited to the child; you must substitute something else, and that right speedily. The mother's milk is, of course, generally the best food for the baby, but not always; the mother may be illy nourished herself—her milk lacking the necessary amount of nutriment to satisfy the hunger of the child. Next to it is the cow's milk; but for young babies is too rich, and should be diluted one half with water and sweetened with white sugar. Should the baby not thrive upon this, add more milk, or secure goat's milk. Should the food still disagree—and you can readily learn the fact by examining the napkin, which should *not* be offensive, neither should it contain any particles of curdled milk, indicating that all had not been digested—then change again—take fresh cow's milk and water, of each a half pint, and graham flour, one teaspoonful; place it in a high tin vessel, which must be set in a kettle of hot water, then boil for half an hour and sweeten. This will be found to be an excellent food for colicy and constipated babies. If found too rich, dilute with more water and add sugar. Very frequently one cow's milk will agree better with a baby than another's—some require richer food than others. Many ignorant nurses stuff babies with all manner of *thick* food when they cry after eating, saying that the "dinner don't satisfy," and "baby must have something richer to eat." They forthwith cram their little stomachs with "pap," farina, beef-tea, soup, and mashed potato. Such food will pass from baby undigested, and will cause colic and diarrhœa; in this condition it will eat eagerly of every and anything that is offered it. A good dose of castor-oil would be better, as the hunger is caused by pain in the bowels from overdistention with undigested food. When a cathartic is necessary, castor-oil is the best that can be given. For a baby of three months, half a teaspoonful of the oil mixed with as much warm molasses, will be readily taken by the babe, and will be a proper dose.

Remember that babies require drink, and that milk does not satisfy thirst. Give your little one a teaspoonful of cold water occasionally and see how readily and eagerly it will take it.

A baby should be bathed three times a week—not oftener. Luke warm water should be used, and the room warm. After carefully drying the little fellow, allow him to lay for a few minutes upon your lap by the stove—he will enjoy the freedom from the entanglement of clothes, and kick up his heels and crow accordingly, and you will so give him an opportunity for exercise of muscles and joints. Finally, mothers, oversee the management of your babies *yourselves* occasionally, and do not trust too implicitly in the discretion of your nurses.

